# Skin Microbiota of the Captive Giant Panda (*Ailuropoda Melanoleuca*) and the Distribution of Opportunistic Skin Disease-Associated Bacteria in Different Seasons

**DOI:** 10.3389/fvets.2021.666486

**Published:** 2021-07-05

**Authors:** Xiaoping Ma, Gen Li, Chao Yang, Ming He, Chengdong Wang, Yu Gu, Shanshan Ling, Sanjie Cao, Qigui Yan, Xinfeng Han, Yiping Wen, Qin Zhao, Rui Wu, Junliang Deng, Zhicai Zuo, Shumin Yu, Yanchun Hu, Zhijun Zhong, Guangneng Peng

**Affiliations:** ^1^Key Laboratory of Animal Disease and Human Health of Sichuan Province, College of Veterinary Medicine, Sichuan Agricultural University, Chengdu, China; ^2^Bioengineering Department, Sichuan Water Conservancy Vocational College, Chengdu, China; ^3^China Conservation and Research Center for the Giant Panda, Chengdu, China; ^4^College of Life Sciences, Sichuan Agricultural University, Chengdu, China

**Keywords:** giant panda (*Ailuropoda melanoleuca*), skin microbiome, 16S rRNA gene, high-throughput sequencing, seasonality

## Abstract

The giant panda is one of the rarest animals in the world. Skin diseases seriously endanger the health of giant panda and are considered the second major cause of its morbidity. Skin microbiota is a complex ecosystem, and the community structure and the pathogenic potential of bacteria on giant panda skin remain largely unclear. In order to understand the skin bacterial flora of captive giant pandas, the microbiota in giant panda skin samples collected during different seasons was profiled *via* 16S *rRNA* gene sequencing. In total, 522 genera from 53 bacterial phyla were detected, with Proteobacteria (40.5%), Actinobacteria (23.1%), Firmicutes (21.1%), Bacteroidetes (9.5%), Cyanobacteria (2.1%), and Thermi (1.2%) as the predominant phyla and *Streptococcus* (13.9%), *Acinetobacter* (9.2%), *Staphylococcus* (2.9%), *Pseudomonas* (5.9%), *Dermacoccus* (4.8%), *Brachybacterium* (2.9%), *Escherichia* (2.7%), *Chryseobacterium* (2.1%), *Arthrobacter* (1.6%), *Kocuria* (1.5%), *Psychrobacter* (1.2%), *Deinococcus* (1.1%), and *Flavobacterium* (1.1%) as the predominant genera. The results indicated that the diversity was lower in winter than in other seasons and higher in autumn than in other seasons, and the abundance in spring was significantly higher than that in other seasons. Several skin disease-associated bacteria were detected as opportunists in the skin microbiota of healthy giant pandas. In this study, the results indicated that the high diversity and abundance of the skin bacteria may have enhanced the occurrence of skin disease in autumn and spring and that skin disease-associated bacteria are the normal components of the skin microbiota.

## Introduction

The giant panda is one of the rarest animals in the world (1). The skin of the giant panda hosts millions of bacteria, fungi, and viruses that make up its skin microbiome. Skin microbes play a vital role in preventing invading pathogens (2). The outermost layer of the skin consists of a stratum corneum, which is rich in lipids and proteins, with hair follicles and glands interspersed among them. These hair follicles and glands secrete lipids, antibacterial peptides, enzymes, salts, and many other compounds (3). Protecting the host from invasion of pathogenic microorganisms is one of the most important functions of the skin (4). Although most microorganisms living on the skin are harmless, or even beneficial, some resident microorganisms are potentially pathogenic under certain conditions (5). However, sufficient knowledge regarding the community structure and pathogenic potential of bacteria on giant panda skin is currently lacking. Skin disease is a refractory disease that mainly damages the skin and hair of the giant panda, thereby affecting its growth and appearance. It reduces the immunity of the giant panda and increases its morbidity and mortality due to other diseases (6).

The gut microbial diversity of giant pandas has been previously described (7). However, the microbial community structures of giant panda skin and fur are yet to be fully researched. Studies that have been conducted on the skin microbial community of giant pandas so far have focused mainly on traditional culture-based methods. Several bacteria and fungi have been found to be conditionally associated with skin disease of giant pandas, including *Cladosporium cladosporioides* (8), *Pestalotiopsis hainanensis* (9), *Microsporum gypseum* (6), *Streptococcus*, and *Staphylococcus* (10). Traditionally, skin microbial communities have been explored using culture methods. However, because culturing selects microbes that thrive under artificial growth conditions, it underestimates the overall diversity of the community (11). Therefore, in order to avoid bias due to culturing and to capture the complete diversity of microbiomes, researchers use more advanced next-generation sequencing and bioinformatics, which are culture-independent methods (ribosomal DNA sequencing) that are widely used to characterize the microbiota of both humans and animals (12, 13). These original sequencing methods use sequence variations in conservative taxonomic markers as molecular fingerprints to identify members of microbial communities. Generally, 16S ribosomal RNA (*rRNA*) genes are used for bacteria (14).

In order to investigate the bacterial flora inhabiting captive giant panda skin, the bacterial flora of giant panda skin samples collected during different seasons were analyzed *via* 16S *rRNA* gene sequencing. This study is the first to reveal the diversity of skin bacterial communities in captive giant pandas. It described the bacterial flora on giant pandas during different seasons and discussed the possible effects of these flora on the pandas, which provided insights into controlling giant panda skin disease.

## Materials and Methods

### Sample Collection

Samples were collected from clinically healthy giant pandas (24 females and 23 males) at the China Conservation and Research Center for the Giant Panda (Ya'an, China). The pandas were housed in dozens of independent enclosures on a mountain where an environment similar to that of wild pandas with heavy broad-leaved forests, green bamboos, and thorns was maintained. Each enclosure, which included an open outdoor area and a closed indoor area, housed one or two giant pandas. The pandas were free to move around indoors and outdoors in their own enclosures, but rarely encountered pandas from other enclosures. All pandas were fed a diet of ~10% steamed cornbread and fruits and 90% bamboo shoots and allowed access to drinking water *ad libitum*. Samples were collected from the upper thoracic limb, head, or dorsum *via* manually scraping appropriate amounts of hair and dander from the skin surface of an ~5.0 × 5.0 cm area, following the removal of most hair. Sampling spots were located on the front part of the body and the surface layer of the skin was avoided, in order to minimize the artificial impact caused by the environment on the skin microbiota. The personnel who performed the sampling wore sterile protective clothing, hats, masks, and latex gloves. Samples were quickly placed in sterile plastic sample bags and shipped to the laboratory on ice within 2 h and stored in a −80°C freezer. Sampling was conducted in March, June, September, and December, representing the four seasons of spring, summer, fall, and winter, respectively. In total, 9 samples were collected for spring, 10 samples were collected for summer, 18 samples were collected for autumn, and 10 samples were collected for winter (Supplementary Table 1).

### DNA Extraction, PCR, and HiSeq Sequencing

Total genomic DNA was extracted from samples using the CTAB/SDS method (15). The V4 regions of 16S *rRNA* genes from all 47 samples were amplified with specific primers (16S V4: 515F: 5′-GTGCCAGCMGCCGCGGTAA-3′, 806R: 5′-GGACTACHVGGGTWTCTAAT-3′), using a Phusion® High-Fidelity PCR Master Mix kit (New England Biolabs, Ipswich, MA, USA). Sequencing libraries were generated and barcoded using a TruSeq® DNA PCR-Free Sample Preparation Kit (Illumina, San Diego, CA, USA) following the instructions of the manufacturer. Quality of the library was assessed *via* a Qubit@ 2.0 Fluorometer (Thermo Scientific, Waltham, MA, USA) and an Agilent Bioanalyzer 2100 system. The amplicons were sequenced on an Illumina HiSeq 2500 platform with 250-bp paired-end reads.

### Data Analysis

Adapters and low-quality reads were removed by preprocessing raw reads according to the quality control process of QIIME tags, using the following procedures (16): filtered read pairs were merged using FLASH (V1.2.7, http://ccb.jhu.edu/software/FLASH/) (17); OTUs were assigned at 97% sequence similarity using Uparse software (Uparse v7.0.1001, http://drive5.com/uparse/) (18); and they were annotated against the Silva Database (http://www.arb-silva.de/) (19) using the RDP classifier algorithm (Version 2.2, http://sourceforge.net/projects/rdp-classifier/) (20). Subsequent analysis of alpha diversity and beta diversity was performed using QIIME (Version 1.9.1) and displayed *via* R software (Version 2.15.3). Linear discriminant analysis coupled with effect size (LEfSe) was performed to identify bacterial taxa differentially represented between seasons at different levels (21).

### Ethics Statement

The sample collection and all the experiments were performed in a style to minimize risk to the giant pandas. All experimental protocols of this study were approved by the Sichuan Agricultural University Animal Ethics Committee and China Conservation and Research Center for the Giant Panda Animal Ethics Committee.

## Results

### Overview of the Sequencing Data

**Figure 1:**
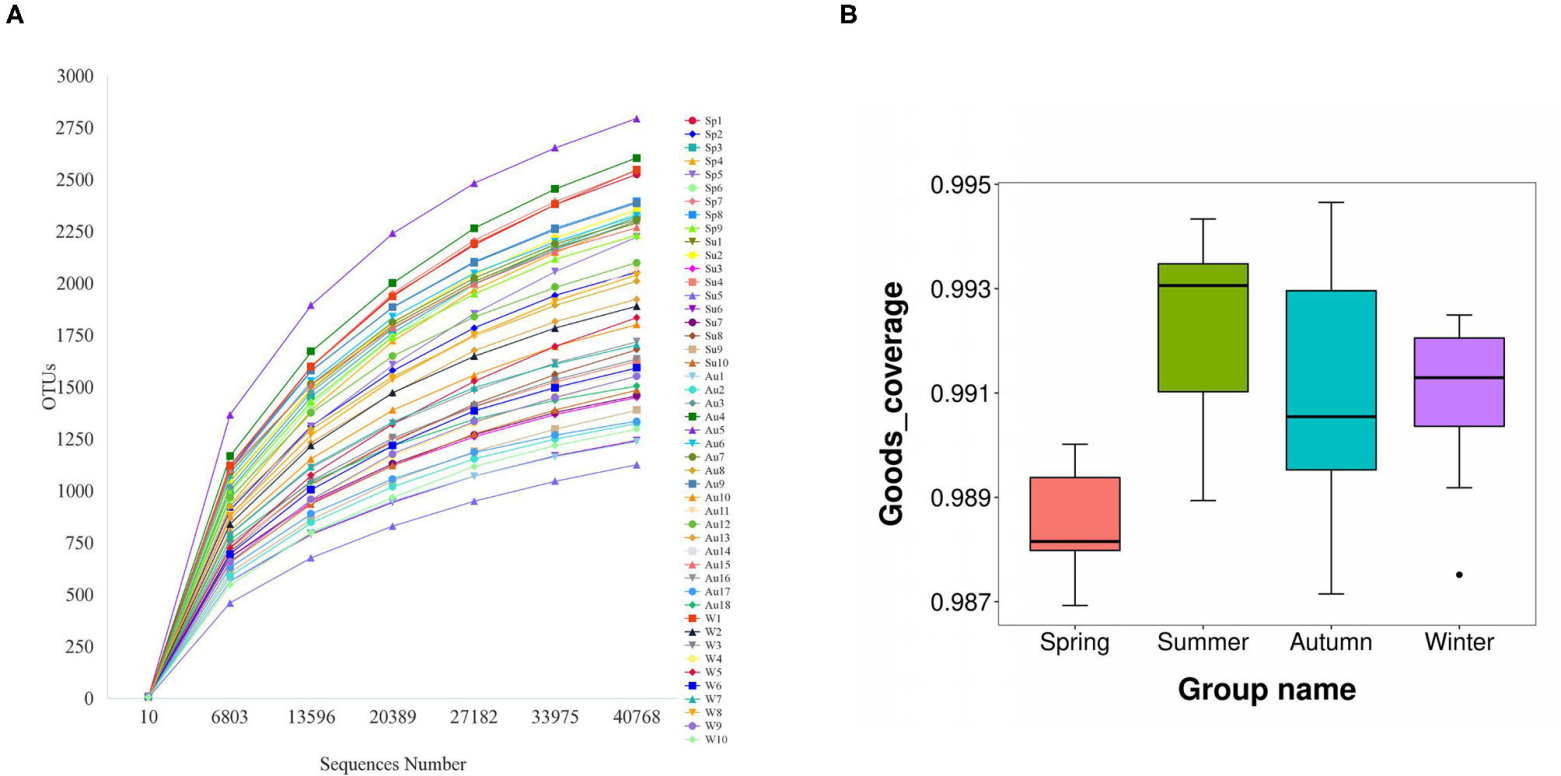
The rarefaction curves started to flatten at a sequence number of 40,000 **(A)**. Good's coverage index estimated that 97.7–99.2% of all species were represented in each sample **(B)**.

The amplicons of the 16S rRNA V4 region of 47 samples were sequenced using the Illumina platform. After quality filtering, 2,531,535 reads of 16S *rDNA* were obtained, and the reads for each sample were between 40,763 and 66,561. Filtered reads were then subjected to taxonomic classification. Following the removal of singleton OTUs (operational taxonomic units), a total of 8,818 OTUs were obtained from all samples and 1,125–2,794 OTUs were identified for each sample. The samples were then rarefied to 40,763 reads for the subsequent analysis of alpha diversity and beta diversity. The rarefaction curves started to flatten at 6,803 sequences (Figure 1A). Good's coverage index (the proportion of non-singleton OTUs in the dataset as a measure of overall OTU sampling completion was used to assess the adequacy of sampling) estimated that 97.7–99.2% of the total species were represented at the rarefied depth in each sample (Figure 1B). These results indicated that the sequencing depth was sufficient to represent the diversity in each sample.

### Skin Microbiota of Giant Panda Was Associated With the Seasons

**Figure 2:**
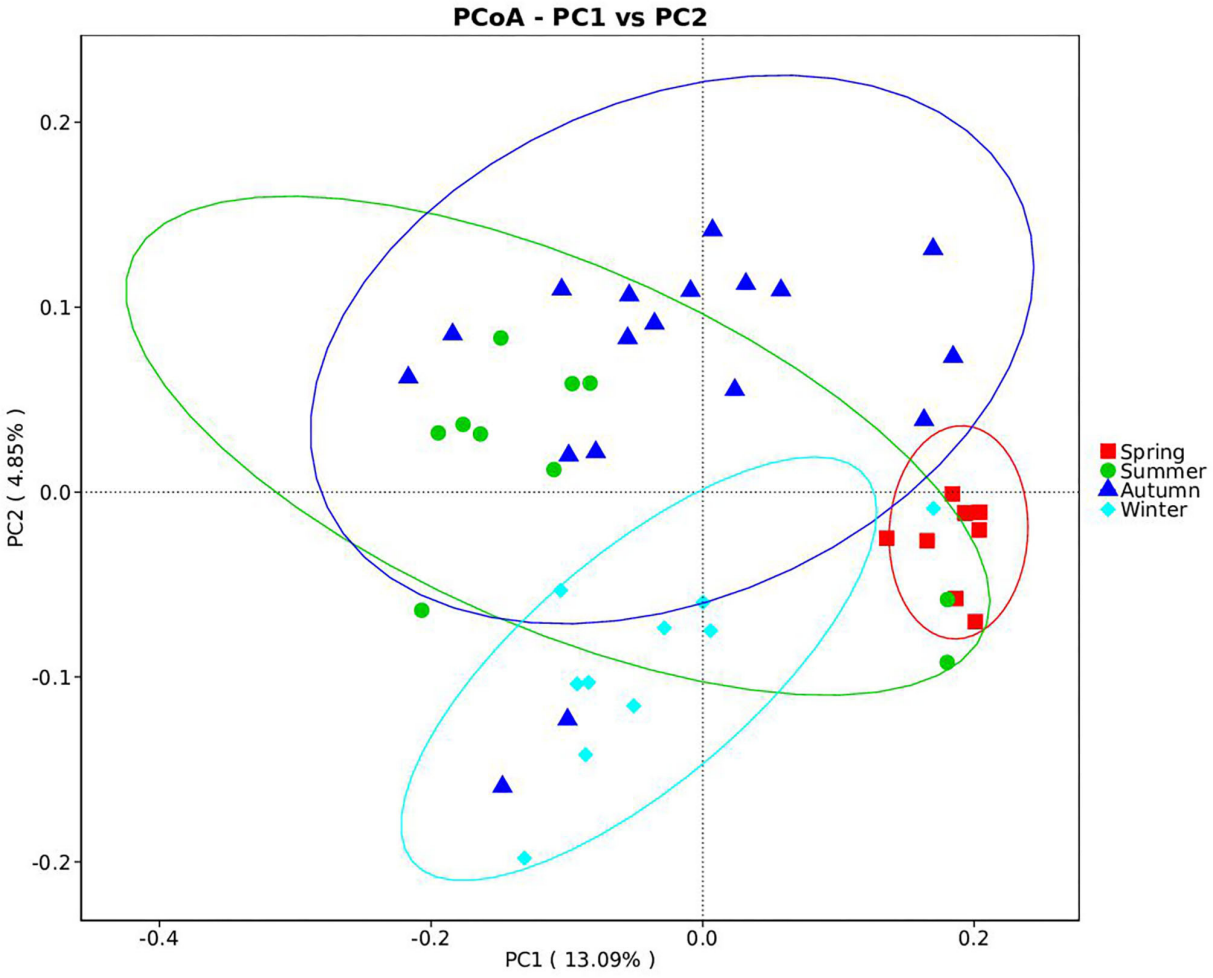
Beta diversity of giant panda (*Ailuropoda melanoleuca*) bacterial skin microbiota from different seasons. The principal coordinate analysis based on unweighted UniFrac metrics indicates that giant panda (*A. melanoleuca*) skin bacterial microbiota is associated with seasons. The close clustering of the samples from each season demonstrates the high phylogenetic similarities of the respective microbiota.

**Figure 3:**
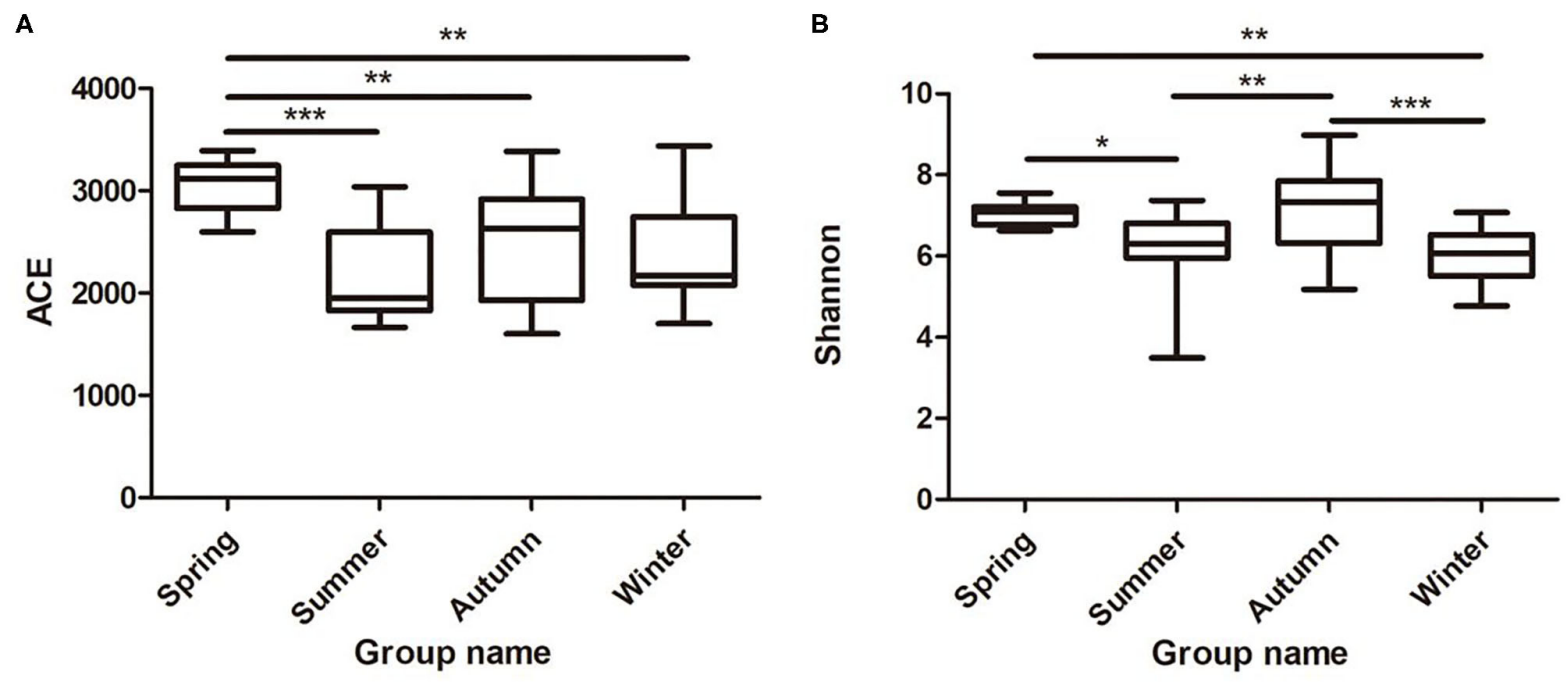
Species richness and diversity of giant panda (*A. melanoleuca*) skin bacterial microbiota measured by 16S rDNA sequencing. Comparison of alpha diversity between seasons are shown in **(A)** (ACE) and **(B)** (Shannon), respectively (Wilcoxon rank-sum test; *p < 0.05, **p < 0.01, ***p < 0.001).

Samples were grouped *via* a beta diversity matrix. PCoA analysis, based on unweighted UniFrac distance metrics of beta diversity, revealed that samples clustered together according to seasons (Figure 2). ANOSIM analysis of the samples from different seasons showed that a skin microbiome corresponding to one season was significantly different from those corresponding to other seasons (Supplementary Table 2), indicating that the skin microbiomes of giant pandas are associated with the seasons. Multiple algorithms such as ACE (abundance-based coverage estimator, a non-parametric asymptotic species richness estimator, to estimate the number of missing species in a sample; the higher values of the ACE estimator indicate higher community richness) and Shannon (entropy estimator, taking into account the species richness and evenness of the community, which varied from zero for communities with a single taxon; the higher value of the Shannon index indicates higher community diversity) were used to estimate the alpha diversity (Figures 3A,B). The ACE estimator was the highest in samples from spring, followed by autumn, winter, and summer (Figures 3A,B). However, although differences between the samples from autumn, winter, and summer were not significant, samples from the other three seasons were all significantly different from the samples from spring (Figures 3A,B). The Shannon index was the highest in the samples from autumn, followed by spring, summer, and winter, in that order (Figures 3A,B). Again, although differences between the samples from autumn, winter, and summer were not significant, samples from the three seasons were all significantly different from the samples from spring (Figures 3A,B). These results indicated that species diversity in winter was lower than that in other seasons, while that in autumn was higher than that in other seasons. Species richness in spring was significantly higher than that in other seasons.

### The Overall Bacterial Community Structure

**Figure 4:**
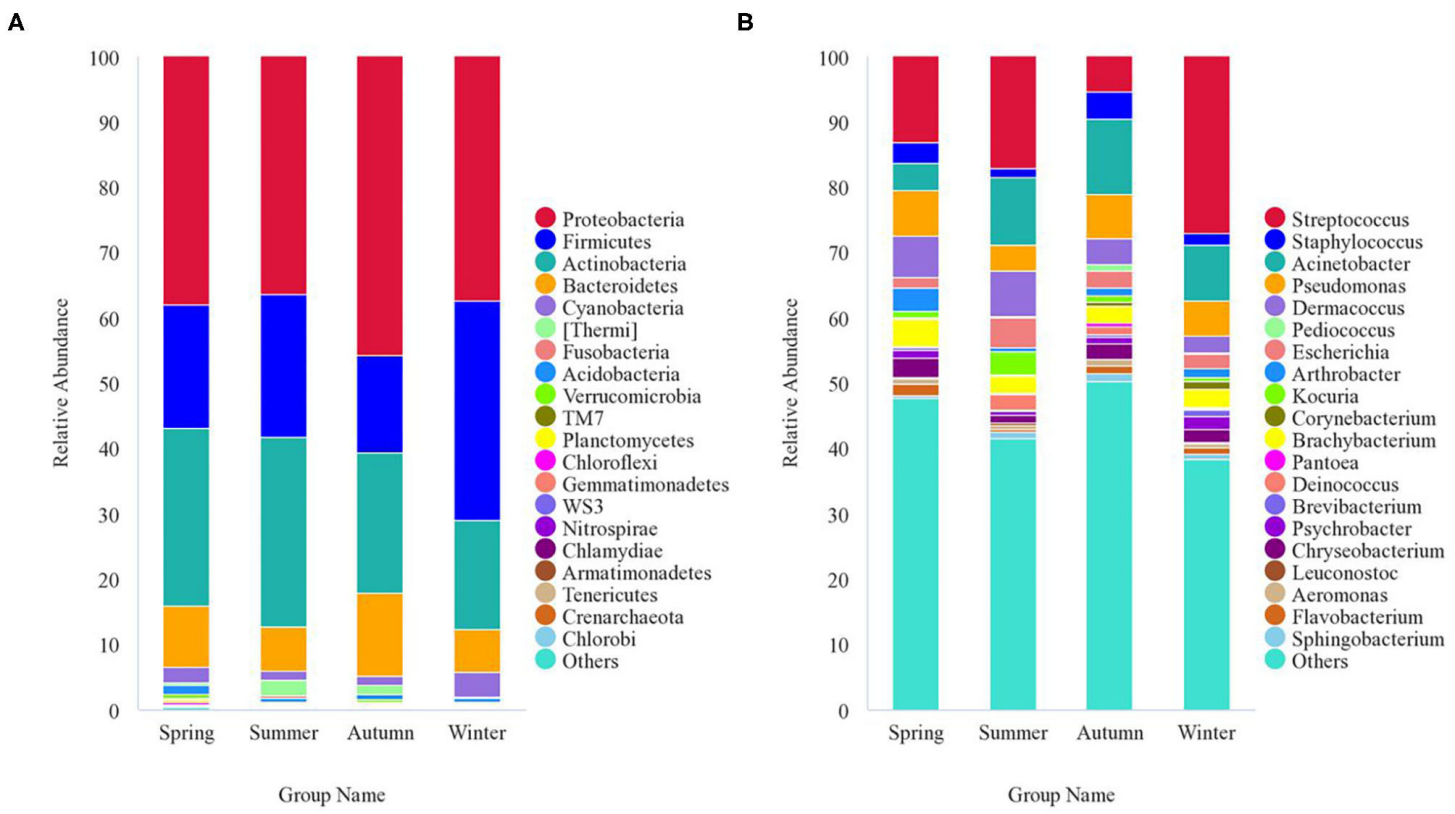
Stacked bar plots showing average percentages of giant panda (*A. melanoleuca*) skin bacterial populations from different seasons. **(A)** Skin bacterial microbiota composition at the phyla level (top 20). **(B)** Skin bacterial microbiota composition at the genera level (top 20).

The 8,818 OTUs obtained from all 47 samples were classified into 53 phyla, 139 classes, 243 orders, 312 families, and 522 genera (Supplementary Table 3). At the phylum level, Proteobacteria (40.5%), Firmicutes (21.1%), Actinobacteria (23.1%), Bacteroidetes (9.5%), Cyanobacteria (2.1%), and Thermi (1.2%) were the predominant taxa (>1%) in all samples, on average (Figure 4A). At the genus level, *Streptococcus* (13.9%), *Staphylococcus* (2.9%), *Acinetobacter* (9.2%), *Pseudomonas* (5.9%), *Dermacoccus* (4.8%), *Brachybacterium* (2.9%), *Escherichia* (2.7%), *Chryseobacterium* (2.1%), *Arthrobacter* (1.6%), *Kocuria* (1.5%), *Psychrobacter* (1.2%), *Deinococcus* (1.1%), and *Flavobacterium* (1.1%) were the predominant genera (>1%) in all samples, on average (Figure 4B). The relative abundance of the taxa varied across different seasons. With respect to the predominant phyla, Proteobacteria (38%) was the main phylum, followed by Actinobacteria (27.1%), Firmicutes (18.8%), Bacteroidetes (9.5%), Cyanobacteria (2.4%), and Acidobacteria (1.4%) in the spring; Proteobacteria (36.3%) was the main phylum, followed by Actinobacteria (29%), Firmicutes (22%), Bacteroidetes (6.8%), Thermi (2.5%), and Cyanobacteria (1.3%) in the summer; Proteobacteria accounted for 45.8%, followed by Actinobacteria (21.5%), Firmicutes (14.8%), Bacteroidetes (12.7%), Cyanobacteria (1.5%), and Thermi (1.3%) in the autumn; and Proteobacteria (37.5%) was the main phylum, followed by Firmicutes (33.5%), Actinobacteria (16.7%), Bacteroidetes (6.6%), and Cyanobacteria (3.6%) in the winter. With respect to the predominant genera, the genera showing a relative abundance of more than 1% were *Streptococcus* (13.1%), *Pseudomonas* (6.9%), *Dermacoccus* (6.3%), *Acinetobacter* (4.3%), *Brachybacterium* (4.1%), *Arthrobacter* (3.5%), *Staphylococcus* (3.1%), *Chryseobacterium* (3.0%), *Escherichia* (1.7%), *Flavobacterium* (1.7%), *Psychrobacter* (1.3%), and *Kocuria* (1.1%) in the spring; *Streptococcus* (17.1%), *Acinetobacter* (10.2%), *Dermacoccus* (7%), *Escherichia* (4.4%), *Pseudomonas* (3.9%), *Kocuria* (3.5%), *Brachybacterium* (2.5%), *Deinococcus* (2.4%), *Staphylococcus* (1.5%), *Paracoccus* (1.4%), and *Chryseobacterium* (1.2%) in the summer; *Acinetobacter* (11.5%), *Pseudomonas* (6.7%), *Streptococcus* (5.3%), *Staphylococcus* (4.2%), *Dermacoccus* (4.1%), *Brachybacterium* (2.6%), *Escherichia* (2.5%), *Chryseobacterium* (2.2%), *Deinococcus* (1.3%), *Flavobacterium* (1.3%), *Sphingobacterium* (1.2%), *Arthrobacter* (1.1%), *Kocuria* (1.1%), *Psychrobacter* (1.1%), *Pediococcus* (1%), and *Aeromonas* (1.0%) in the autumn; and *Streptococcus* (27.1%), *Acinetobacter* (8.6%), *Pseudomonas* (5.3%), *Brachybacterium* (2.8%), *Dermacoccus* (2.7%), *Escherichia* (2.1%), *Psychrobacter* (2.0%), *Chryseobacterium* (2.0%), *Staphylococcus* (1.6%), *Arthrobacter* (1.4%), *Corynebacterium* (1.3%), *Brevibacterium* (1.1%), *Flavobacterium* (1.1%), and *Clostridium* (1.0%) in the winter.

### Season-Related Genera and Abundance Variation of Bacteria

**Figure 5:**
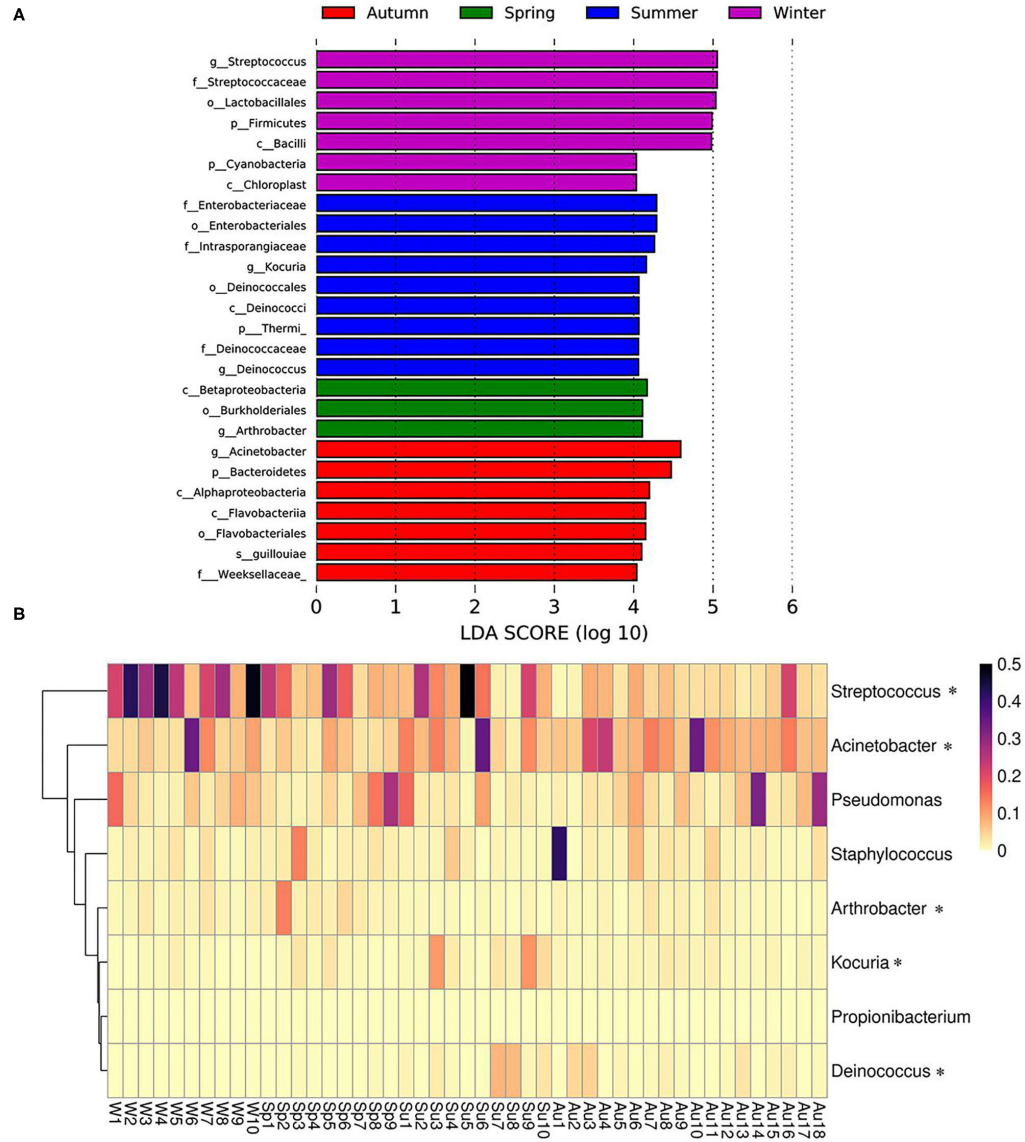
Season-related genera and abundance variation of skin disease-associated bacteria. **(A)** Skin bacteria associated with seasons identified *via* linear discriminant analysis coupled with effect size (LEfSe) using default parameters. **(B)** Heatmap showing the relative abundance of season-related genera (denoted by “*”) and skin disease-associated bacteria (only taxa with a defined genus are shown).

Season-related genera were identified *via* linear discriminant analysis (LDA) and effect size analysis. Five genera were associated with winter (*n* = 1), summer (*n* = 2), spring (*n* = 1), and autumn (*n* = 1), respectively (Figure 5A). *Arthrobacter* was associated with spring, *Kocuria and Deinococcus* were associated with summer, *Acinetobacter* was associated with autumn, and *Streptococcus* was associated with winter (Figures 5A,B). Currently, the reports pertaining to bacterial skin diseases in giant pandas are scant, including *Streptococcus* and *Staphylococcus* (10). However, we detected the presence of related bacteria that may cause skin diseases in humans and animals, including *Streptococcus, Staphylococcus, Acinetobacter, Pseudomonas, Arthrobacter*, and *Propionibacterium* (Figure 5B). *Streptococcus* was more abundant than *Staphylococcus, Acinetobacter, Pseudomonas, Arthrobacter*, and *Propionibacterium*. The average relative abundance of *Streptococcus* was 13.9%, while those of *Staphylococcus, Acinetobacter, Pseudomonas, Arthrobacter*, and *Propionibacterium* were 2.9, 9.2, 5.9, 1.6, and 0.02%, respectively. *Streptococcus, Acinetobacter*, and *Arthrobacter* were season-related, where *Arthrobacter* was more abundant in the spring, *Acinetobacter* was more abundant in the autumn, and *Streptococcus* was more abundant in the winter. However, there were no significant variations between *Pseudomonas* and *Propionibacterium* during different seasons (Figure 5B).

## Discussion

The skin microbiome hosts a wide variety of microorganisms, including bacteria, viruses, and fungi. Skin microbiota protects against harmful microbes, maintains skin homeostasis, and informs our immune system (22). On the other hand, microbial dysbiosis may cause or exacerbate skin diseases. In this study, we profiled skin bacterial flora of captive giant pandas for the first time using 16S rRNA-based NGS sequencing. We detected 522 genera in 53 bacterial phyla. Among these, Proteobacteria, Actinobacteria, and Firmicutes were the predominant phyla, and *Streptococcus, Acinetobacter*, and *Staphylococcus* were the predominant genera. Our results showed that the skin bacterial community of the giant panda differs from that of humans and other animals (canine and feline). Previous studies showed that canine skin was dominated by *Fusobacterium* and *Pseudomonas*, feline skin was dominated by *Porphyromonas* and *Staphylococcus*, and human skin was dominated by *Propionibacterium* and *Staphylococcus* (13, 23, 24). Skin microbiota in different hosts may be affected by genetic differences and pelage characteristics, as well as different hygiene practices and environmental exposures between host species.

The skin bacterial flora of captive giant pandas change with the seasons. Moreover, the bacterial abundance in spring was significantly higher than that in other seasons and the diversity in winter was lower than that in other seasons, and skin bacterial diversity in autumn was higher than that in other seasons. Spring is warm and humid in Sichuan, China. High temperatures compounded by the humid environment of spring are optimal for the growth and reproduction of bacteria, and this may have contributed to the differences seen in abundance. Cold and dry winter conditions may have contributed to the difference in diversity. Interestingly, we found that the diversity and abundance of bacterial communities were not solely related to the temperature and humidity of the environment. For example, the ambient temperature is higher in summer, but the Shannon diversity index indicated that microbial diversity in samples collected during summer was not higher than that in samples collected during spring and autumn. The ACE index showed that microbial abundance in samples collected in summer was lower than that in samples collected in other seasons. However, other studies have reported different results, and studies of the human skin microbiome show that despite exposure to the external environment, the bacterial, fungal, and viral communities in the skin were largely stable over time (12). A study of the skin microbiota in healthy dogs showed that temporality (season of birth and time spent in the kennel) affected all skin sites (13). The stability of the skin microbiome seems to vary between species over time. Changes in the skin microbiome are not just related to changes in ambient temperature and humidity, but also to the state of immune activation, host genetic predisposition, barrier status, microbe localization, and microbe–microbe interactions (3). This may be because animals interact with their living environment and are constantly exposed to soil, water, plants, and sewage. Therefore, the skin microbiome of animals is prone to change with seasonal changes. Humans generally live in a relatively stable environment, due to which changes in the skin microbiome are not obvious. At the genus level, five genera were significantly more abundant during the specific seasons. *Arthrobacter* was associated with spring, *Kocuria* and *Deinococcus* were associated with summer, *Acinetobacter* was associated with autumn, and *Streptococcus* was associated with winter. These bacterial taxa have been isolated from the environment as well as animals and physiologically characterized (25–32). *Streptococcus* is a common resident of human skin. *Streptococcus pyogenes* is a very important human pathogen that is commonly associated with skin or throat infections, but may also cause life-threatening conditions including sepsis, streptococcal toxic shock syndrome, and necrotizing fasciitis (33). An increase in the abundance of *Streptococcus* in winter may increase the possibility of panda skin being infected by *Streptococcus*. *Acinetobacter* is a common bacterium found in human and animal skin (29, 34). These bacteria, which have already been recognized as important nosocomial pathogens in humans, are becoming increasingly recognized in opportunistic pathogens of animals. *Acinetobacter baumannii* is a gram-negative skin pathogen. *A. baumannii* causes a variety of infections, most of which involve the respiratory tract, although bacteremia and skin wound infections have also been reported (35, 36). There are no case reports of skin diseases caused by *A. baumannii* in giant pandas. *Arthrobacter, Kocuria*, and *Deinococcus* are common commensal bacteria found on human skin, but occasionally, infections have been reported (37–40).

Microbiota of giant pandas also carries other taxa, including *Staphylococcus, Pseudomonas*, and *Propionibacterium*, that are known to cause bacterial skin diseases in humans and other animals. However, the abundance of these taxa does not change significantly with the seasons. *Staphylococcus*, although generally identified as a commensal, commonly causes human bacterial infections of the skin and other soft tissues, bones, bloodstream, and the respiratory tract (41). *Staphylococcus* is responsible for most bacterial skin infections and can initiate or exacerbate skin disorders in the broader context of barrier defects or altered immunity. It has been reported in an infection case of an aged giant panda, causing pressure ulcers on the body surface (10). *Pseudomonas* is a gram-negative bacterium, and *Pseudomonas aeruginosa*, which is most often associated with opportunistic infections, can also appear in healthy individuals. *P. aeruginosa* infections range from local skin infections to life-threatening diseases (42). The gram-positive anaerobic bacterium *Propionibacterium acnes* is part of the normal microbiome of human skin and mucosal surfaces. Although *P. acnes* is commonly associated with skin health, it is also an opportunistic pathogen associated with a range of human infections and clinical conditions (43). In the present study, it was found that potentially pathogenic bacteria were ubiquitous across all samples. Bacterial skin diseases in giant pandas are mainly reported as staphylococcal and streptococcal infections. Clinically, aged pandas and pandas with low immunity are prone to skin diseases (10). Commensal bacteria of the skin, mucosa, or gastrointestinal tract, including staphylococci and *P. acnes*, are often opportunistic pathogens. It is important to study potential skin pathogens. The development of bacterial skin disease is multifactorial and may not only be associated with the bacterial community structure but also host skin integrity and body immunity as well as associated with the fungal and viral communities of skin surfaces. However, the high diversity and abundance of the skin bacteria observed in this study during the spring and autumn may increase the risk of the bacterial pathogens infecting the host. In addition, the fact that captive pandas monitored in this study were all healthy may have limited our investigation of pathogenic bacteria inhabiting the skin of captive pandas. Thus, applying metagenomics technology to study captive panda skin microbiomes in the future may enable a more complete understanding of the captive panda skin microbiota.

## Conclusion

In this study, the skin microbiota of healthy captive giant pandas was profiled using 16S rRNA-based NGS sequencing. The results indicated that the skin microbiota of giant pandas changes with the seasons and that the diversity and abundance of skin bacteria was the highest in autumn, followed by spring. This indicates that the risk of bacterial skin diseases in giant pandas may increase in autumn and spring. Several skin disease-associated bacteria were detected in the skin microbiota of healthy giant pandas, indicating that skin disease-associated bacteria are normal components of the skin microbiota of giant pandas and may cause bacterial skin diseases, conditionally. It is essential for us to have a better understanding of the microbial population that lives on the skin of giant pandas, to be able to describe the skin microbiota of healthy pandas, identify changes in the skin microbiota that occur in a disease state, and potentially identify better measures to treat skin conditions. The present study, to our knowledge, revealed the skin microbiota of the captive giant pandas for the first time and provided insights into the development of bacterial skin diseases in these endangered animals.

## Data Availability Statement

The datasets generated for this study can be found in the National Center for Biotechnology Information (NCBI). (SRA: https://www.ncbi.nlm.nih.gov/sra/PRJNA688828).

## Ethics Statement

The animal study was reviewed and approved by Sichuan Agricultural University Animal Ethics Committee and China Conservation and Research Center for the Giant Panda Animal Ethics Committee.

## Author Contributions

XM, GL, CY, MH, CW, YG, and SL carried out the sample collections, conceived the study, and drafted the manuscript. GL, SC, QY, XH, YW, QZ, and RW participated in the data analysis. JD, ZZu, SY, YH, ZZh, XM, and GP participated in the study design and coordination and helped draft the manuscript. All authors have read and approved the final manuscript.

## Conflict of Interest

The authors declare that the research was conducted in the absence of any commercial or financial relationships that could be construed as a potential conflict of interest.
