# Supplementary material for: Skin Microbiota of the Captive Giant Panda (Ailuropoda Melanoleuca) and the Distribution of Opportunistic Skin Disease-Associated Bacteria in Different Seasons
Source: Front Vet Sci. 2021 Jul 5;8:666486. doi: 10.3389/fvets.2021.666486 (PMC8286994; doi:10.3389/fvets.2021.666486)
Supplement: Supplementary file 1 [file Data_Sheet_1.zip › Table 1 (5).DOCX]

**Supplemental Table 1** The sample information

| Group | Sample number | sample name | Sampling time |
| --- | --- | --- | --- |
| Spring | Sp1 | zhuang mei | 03.11.2015 |
|  | Sp2 | feng yi | 03.11.2015 |
|  | Sp3 | yuan yuan | 03.11.2015 |
|  | Sp4 | wu jun | 03.11.2015 |
|  | Sp5 | ge ge | 03.11.2015 |
|  | Sp6 | bo yang | 03.11.2015 |
|  | Sp7 | qian qian | 03.11.2015 |
|  | Sp8 | shu qin | 03.11.2015 |
|  | Sp9 | xi mei | 03.11.2015 |
| Summer | Su1 | zhuang mei | 06.11.2015 |
|  | Su2 | feng yi | 06.11.2015 |
|  | Su3 | yuan yuan | 06.11.2015 |
|  | Su4 | wu jun | 06.11.2015 |
|  | Su5 | ge ge | 06.11.2015 |
|  | Su6 | bo yang | 06.11.2015 |
|  | Su7 | qian qian | 06.11.2015 |
|  | Su8 | shu qin | 06.11.2015 |
|  | Su9 | xi mei | 06.11.2015 |
|  | Su10 | yi bao | 06.11.2015 |
| Fall | Au1 | zhuang mei | 09.11.2015 |
|  | Au2 | feng yi | 09.11.2015 |
|  | Au3 | yuan yuan | 09.11.2015 |
|  | Au4 | wu jun | 09.11.2015 |
|  | Au5 | ge ge | 09.11.2015 |
|  | Au6 | bo yang | 09.11.2015 |
|  | Au7 | qian qian | 09.11.2015 |
|  | Au8 | shu qin | 09.11.2015 |
|  | Au9 | xi mei | 09.11.2015 |
|  | Au10 | yi bao | 09.11.2015 |
|  | Au11 | lu lu | 09.11.2015 |
|  | Au12 | su shan | 09.11.2015 |
|  | Au13 | an an | 09.11.2015 |
|  | Au14 | guo guo | 09.11.2015 |
|  | Au15 | wu gang | 09.11.2015 |
|  | Au16 | dou dou | 09.11.2015 |
|  | Au17 | wen yu | 09.11.2015 |
| Winter | W1 | zhuang mei | 12.11.2014 |
|  | W2 | feng yi | 12.11.2014 |
|  | W3 | yuan yuan | 12.11.2014 |
|  | W4 | wu jun | 12.11.2014 |
|  | W5 | ge ge | 12.11.2014 |
|  | W6 | bo yang | 12.11.2014 |
|  | W7 | qian qian | 12.11.2014 |
|  | W8 | shu qin | 12.11.2014 |
|  | W9 | xi mei | 12.11.2014 |
|  | W10 | yi bao | 12.11.2014 |

Note:The same giant panda (*A. melanoleuca*） is marked in blue or green
