# Supplementary material for: Skin Microbiota of the Captive Giant Panda (Ailuropoda Melanoleuca) and the Distribution of Opportunistic Skin Disease-Associated Bacteria in Different Seasons
Source: Front Vet Sci. 2021 Jul 5;8:666486. doi: 10.3389/fvets.2021.666486 (PMC8286994; doi:10.3389/fvets.2021.666486)
Supplement: Supplementary file 1 [file Data_Sheet_1.zip › Table 2 (2).DOCX]

**Supplemental Table 2** Anosim analysis of the samples between different seasons

| Group | R-value | P-value |
| --- | --- | --- |
| Winter-Autumn | 0.4748 | 0.001 |
| Summer-Autumn | 0.2362 | 0.008 |
| Summer-Winter | 0.3727 | 0.001 |
| Spring-Autumn | 0.3197 | 0.002 |
| Spring-Winter | 0.3377 | 0.004 |
| Spring-Summer | 0.39 | 0.001 |
