# Supplementary figures and images for: Skin Microbiota of the Captive Giant Panda (Ailuropoda Melanoleuca) and the Distribution of Opportunistic Skin Disease-Associated Bacteria in Different Seasons
Source: Front Vet Sci. 2021 Jul 5;8:666486. doi: 10.3389/fvets.2021.666486 (PMC8286994; doi:10.3389/fvets.2021.666486)

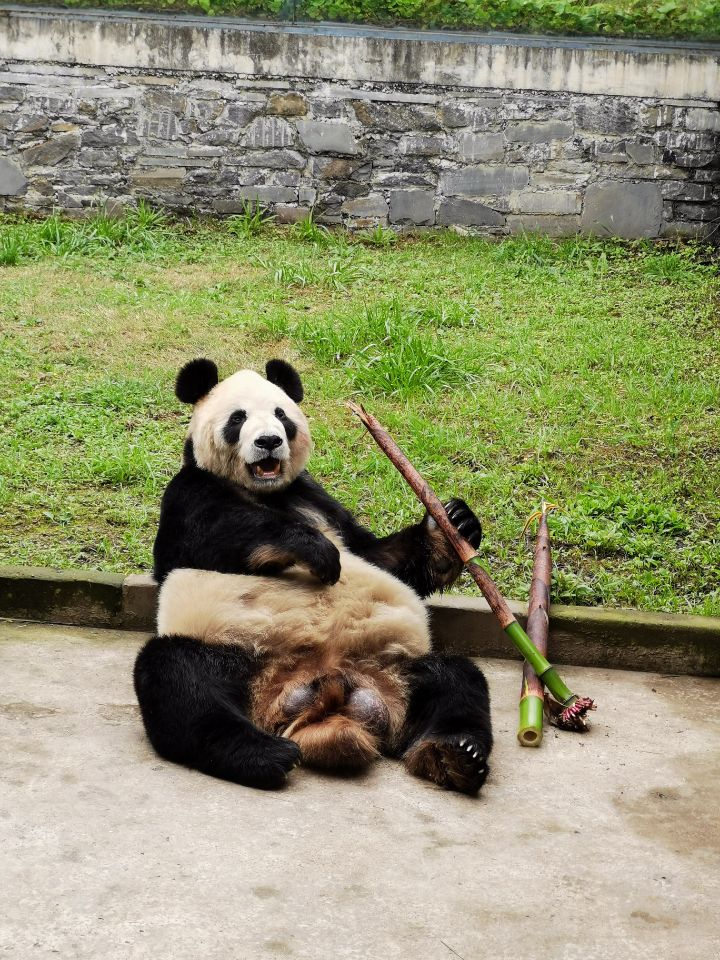

Supplement: Supplementary file 2 [file Image_1.JPEG]

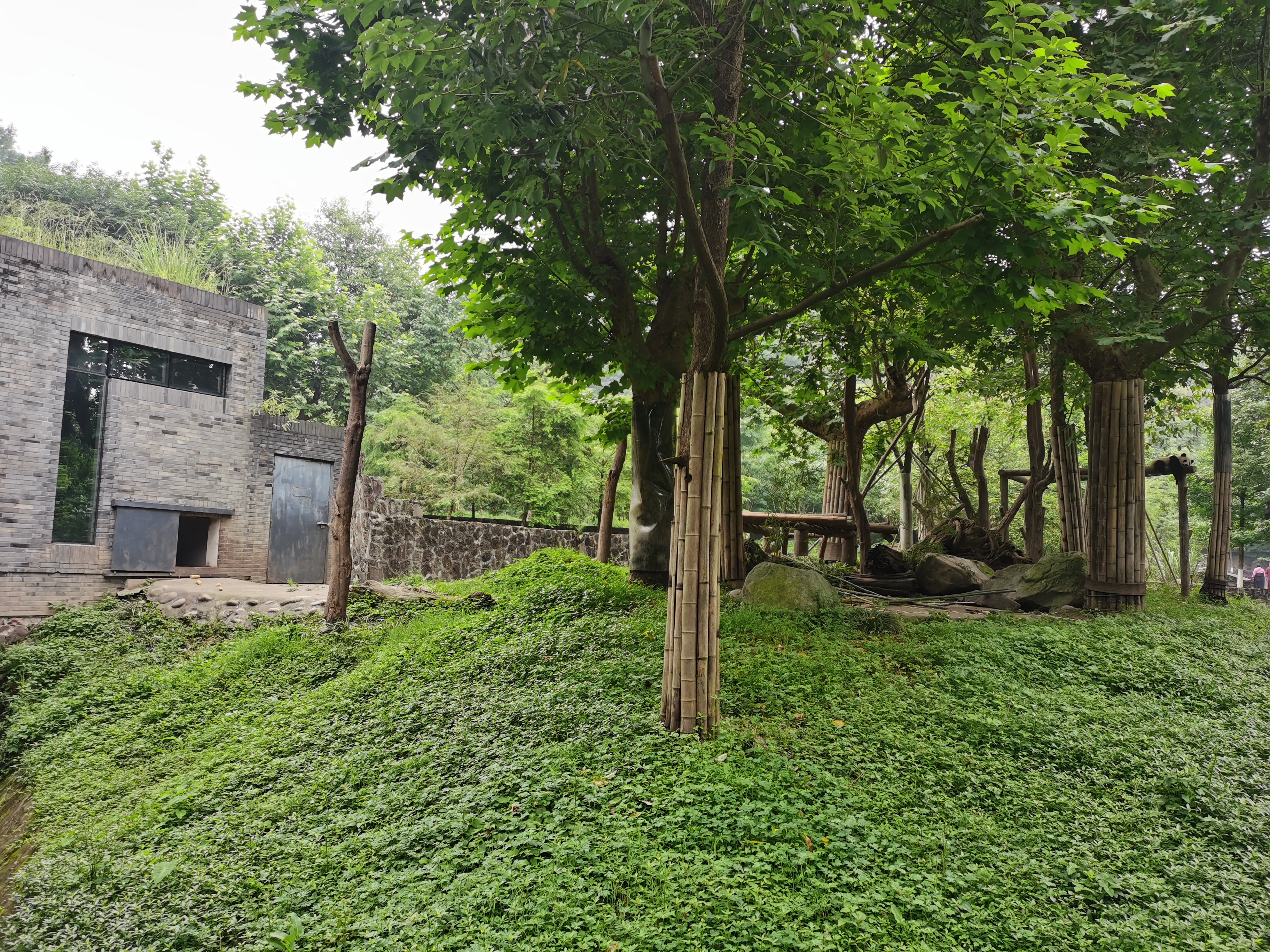

Supplement: Supplementary file 3 [file Image_2.JPEG]

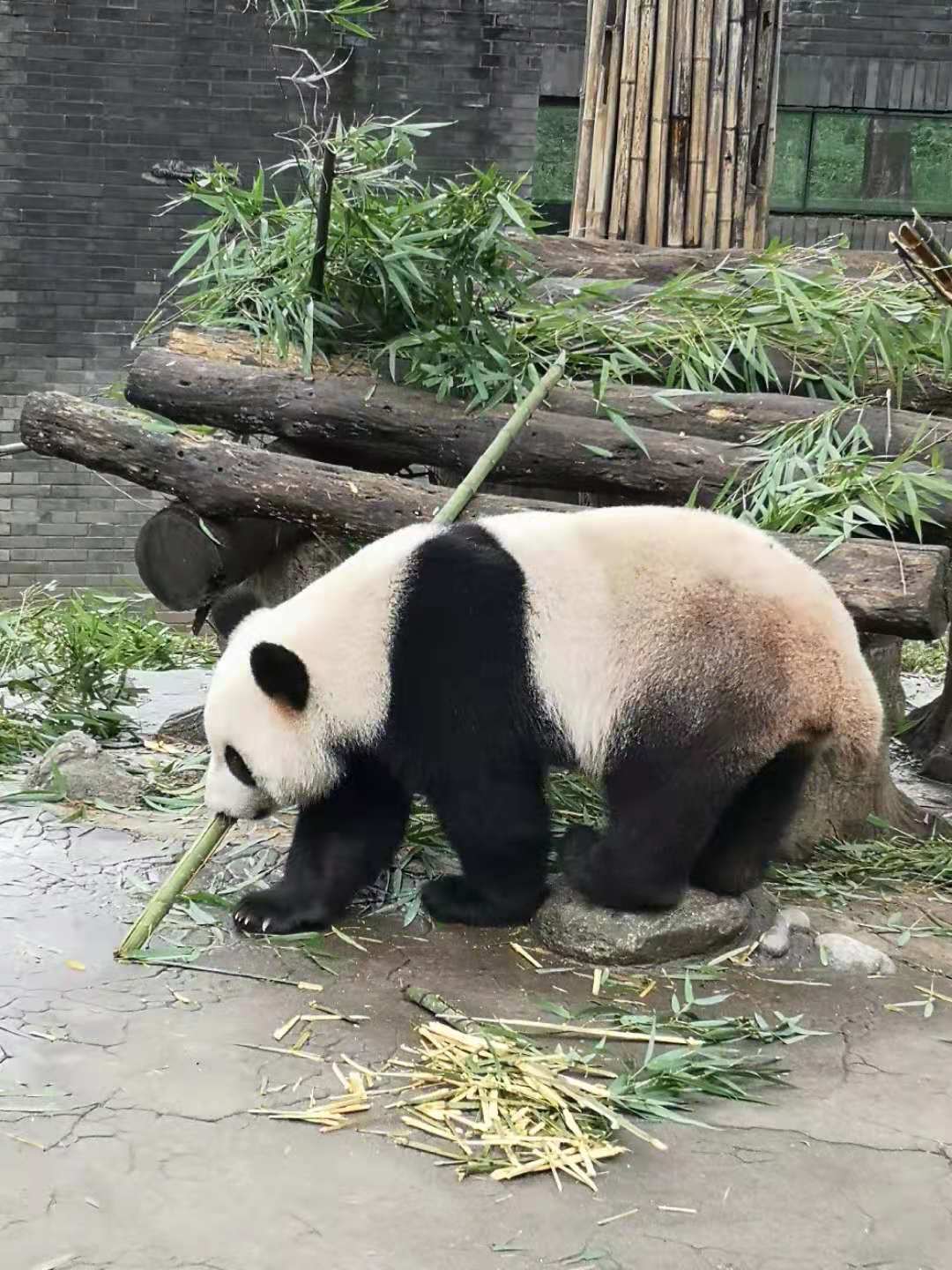

Supplement: Supplementary file 4 [file Image_3.JPEG]
